# Environmental and genetic effects on tomato seed metabolic balance and its association with germination vigor

**DOI:** 10.1186/s12864-016-3376-9

**Published:** 2016-12-19

**Authors:** Leah Rosental, Adi Perelman, Noa Nevo, David Toubiana, Talya Samani, Albert Batushansky, Noga Sikron, Yehoshua Saranga, Aaron Fait

**Affiliations:** 1 The French Associates Institute for Agriculture and Biotechnology of Dryland, the Jacob Blaustein Institutes for Desert Research, Ben-Gurion University of the Negev, Sede Boqer Campus, Midreshet Ben Gurion, 84990 Israel; 2 Robert H. Smith Institute of Plant Sciences and Genetics in Agriculture, The Hebrew University of Jerusalem, Rehovot, 76100 Israel

**Keywords:** Metabolites, Germination, Maternal environment, Tomato introgression lines, Quantitative trait loci, QTL

## Abstract

**Background:**

The metabolite content of a seed and its ability to germinate are determined by genetic makeup and environmental effects during development. The interaction between genetics, environment and seed metabolism and germination was studied in 72 tomato homozygous introgression lines (IL) derived from *Solanum pennelli* and *S. esculentum* M82 cultivar. Plants were grown in the field under saline and fresh water irrigation during two consecutive seasons, and collected seeds were subjected to morphological analysis, gas chromatograph-mass spectrometry (GC-MS) metabolic profiling and germination tests.

**Results:**

Seed weight was under tight genetic regulation, but it was not related to germination vigor. Salinity significantly reduced seed number but had little influence on seed metabolites, affecting only 1% of the statistical comparisons. The metabolites negatively correlated to germination were simple sugars and most amino acids, while positive correlations were found for several organic acids and the N metabolites urea and dopamine. Germination tests identified putative loci for improved germination as compared to M82 and in response to salinity, which were also characterized by defined metabolic changes in the seed.

**Conclusions:**

An integrative analysis of the metabolite and germination data revealed metabolite levels unambiguously associated with germination percentage and rate, mostly conserved in the different tested seed development environments. Such consistent relations suggest the potential for developing a method of germination vigor prediction by metabolic profiling, as well as add to our understanding of the importance of primary metabolic processes in germination.

**Electronic supplementary material:**

The online version of this article (doi:10.1186/s12864-016-3376-9) contains supplementary material, which is available to authorized users.

## Background

Seeds play a major role in agriculture, both as products for human food and animal feed and as plant propagation units. The seed quality for propagation is determined by its potential to germinate and produce viable and robust seedlings [1, 2]. The uniformity and rate of germination are important agronomic traits, especially in crops that are sown directly in the field [3], which are governed by internal mechanisms such as plant hormone levels, transcription regulation [4] and environmental conditions, including water availability, temperature, nitrate levels and light [5–7].

Seed germination is inherently related to seed metabolism, which changes throughout its maturation, desiccation and germination processes [8, 9]. Maturing seeds accumulate transcripts and metabolites necessary for seed germination [10]. During germination, glucose at high levels can support abscisic acid (ABA) signaling, delaying germination and starch degradation in tomato [11] and Arabidopsis [12]. Intermediates of the tricarboxylic acid (TCA) cycle accumulate during seed priming [13], likely in preparation for the high energy demands of germination. Amino acids are also used as energy production sources during the early stages of germination via various pathways [2, 14]. Cell wall metabolism is essential for the loosening of the endosperm cap in tomato and for the elongation of the radicle leading to germination [15]. Despite these studies, the understanding of the relation between primary seed metabolism and germination is still poor [16]. Fundamental questions remain unanswered, including: what are the metabolic processes required to enable or boost germination and seedling establishment? Therefore, an integrated view of the existing degree of variability in the metabolite profile of seeds is necessary. High-throughput methods, such as gas chromatography coupled to a mass spectrometer (GC-MS) [17], combined with multivariate approaches for analyzing mapping populations’ natural diversity, can aid in developing a comprehensive picture of the metabolic network.

The introgression line (IL) population between *Solanum Pennelli* and *S. esculentum,* cultivar M82 [18, 19] has proven to be an excellent tool for researching and identifying QTLs [20], leading to the cloning of agronomically and biologically important genes [21, 22]. In exploring the link between metabolism and plant traits, using the natural variability of the IL population, Schauer et al. [23] identified 889 fruit metabolic QTLs and found that central metabolites were more associated with morphological traits than metabolites related to secondary metabolism. Schauer et al. [24] also studied the mode of inheritance of the tomato fruit’s metabolic traits. They found that metabolite content is affected by environmental and genetic factors, and that metabolites sharing QTLs are probably jointly regulated. In the same IL population, Toubiana et al. [25] identified 30 QTLs likely regulating seed metabolism. The analysis revealed a group of amino acids that were highly co-regulated in association with a group of genes on chromosome 2 of the glycine and serine metabolism [26].

Salinity affects seed germination and crop establishment worldwide, leading to significant reductions in yield and crop quality. Tomato is considered to have a moderate tolerance to salt stress and is affected by salinity starting at a soil extract electrical conductivity (EC) of 2.5–3 dS/m [27]. The effect of salinity on tomato germination has been studied in a wide range of wild species and cultivar accessions [27, 28], and the results show reduced germination percentages and delays in the germination rate. The wild tomato *S. pennelli* has a better tolerance to germination under saline conditions, and several attempts were made to discover the QTLs and wild-type alleles related to this phenotype [29, 30]. While the effects of various environmental conditions during development on germination have been studied [31–34], the mediating effect of parental genetics and growth conditions during seed development on seed metabolism and germination has not been entirely grasped.

By employing seeds collected from the ILs grown in the field under fresh water and a mild salinity, we explored the link between the seed metabolic traits and parental environmental conditions and genetics on modulating seed germination.

## Results

In this study, 72 ILs and their genetic background cultivar M82 were examined for the effect of growth under EC = 1.5 dS/m (control) and EC = 6 dS/m (saline) during two seasons (2010 and 2011) on seed numbers, weight, metabolic content and germination. Seeds developed on plants grown under saline irrigation (SDS) were compared to seeds developed on plants grown in fresh water (SDF), as the control, for all examined traits. Identification of putative QTLs was attained by comparing the ILs to M82 for any given trait.

### Seed weight

The average weight of mature seeds was determined and compared between treatments and lines. No lines had a significant (*p* < 0.01) difference in seed weight between SDS and SDF. There were, however, differences in seed weight among some ILs and M82 (Table 1). In SDF, five ILs (**IL1-1-3**, IL2-4, **IL4-3-2**, IL7-2 and IL11-1) had significantly (*p* < 0.01) lower seed weights than M82, and six ILs (IL7-4, **IL7-4-1**, **IL8-2-1**, **IL8-3-1**, IL10-1-1 and **IL11-4-1**) had significantly higher seed weights. In SDS, **IL1-1-3** and **IL4-3-2** had significantly lower seed weights than M82, and eleven ILs (IL1-4-18, IL3-5, **IL7-4-1**, IL7-5-5, **IL8-2-1**, IL8-3, **IL8-3-1**, IL10-3, IL11-4, **IL11-4-1** and IL12-1-1) had seed weights that were significantly higher than M82. Six ILs (noted in bold) had matching significant differences in both treatments, suggesting putative robust QTLs for seed weight. A gene influencing seed size has been isolated from the IL4-3-2 genomic region [35], showing the potential of similar QTL-based research.

**Table 1:** ILs with putative QTLs for seed weight

SDF				SDS			
		Seed				Seed	
		Weight				Weight	
		(mg)	(sd)			(mg)	(sd)
**M82**		2.698	(0.18)	**M82**		2.609	(0.16)
**IL1-1-3**	−	2.162	(0.21)^*^	**IL1-1-3**	−	2.114	(0.14)^**^
IL2-4	−	2.123	(0.09)^***^	IL1-4-18	+	3.138	(0.24)^*^
**IL4-3-2**	−	2.148	(0.06)^***^	IL3-5	+	3.042	(0.14)^**^
IL7-2	−	2.393	(0.07)^**^	**IL4-3-2**	−	2.221	(0.16)^*^
IL7-4	+	3.040	(0.1)^**^	**IL7-4-1**	+	3.192	(0.17)^**^
**IL7-4-1**	+	3.497	(0.21)^*^	IL7-5-5	+	2.880	(0.12)^*^
**IL8-2-1**	+	3.145	(0.2)^*^	**IL8-2-1**	+	3.108	(0.25)^*^
**IL8-3-1**	+	3.239	(0.18)^*^	IL8-3	+	2.982	(0.05)^***^
IL10-1-1	+	3.280	(0.27)^*^	**IL8-3-1**	+	3.218	(0.22)^*^
IL11-1	−	2.090	(0.14)^**^	IL10-3	+	3.069	(0.17)^*^
**IL11-4-1**	+	3.195	(0.15)^**^	IL11-4	+	2.926	(0.06)^***^
				**IL11-4-1**	+	3.211	(0.17)^**^
				IL12-1-1	+	3.357	(0.11)^***^

ILs that had significantly increased or decreased seed weight compared to M82 in season 1 are marked by + or−, respectively. ILs with significant differences under both conditions are presented in bold

Asterisks mark significance levels (five replicates) with the Bcp of ^*^ *p* < 0.05, ^**^ *p* < 0.01, ^***^ *p* < 0.001

A comparison of IL seed weight with M82 between the two seasons revealed consistent trends of variance (Additional File 1: Table S2). Overall, there was considerable overlap in the ILs with significant differences compared to M82 in the four growth conditions of the two treatments and both seasons. Twelve of the 32 putative QTLs detected were confirmed in at least two conditions, and seven of the putative QTLs were shared in all conditions. The general stability in seed weight among the ILs and M82 across environmental factors, such as seasons and salinity treatments, suggests a strong genetically regulated trait that is less affected by the environment. No other physiological or metabolic trait of the seed that was measured in this study displayed a similar stability.

### Seed number and seed abortion

**Fig. 1:**
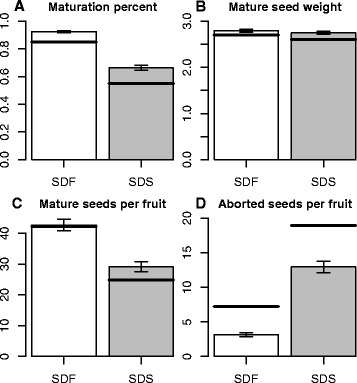
Seed weight and numbers in response to salinity. Whole population maturation percent (**a**), average seed weight in grams (**b**), number of mature seeds per fruit (**c**) and number of aborted seeds per fruit (**d**) are presented by the *bars*. Corresponding average levels of M82 are noted by *black horizontal lines. Error bars*: standard error

In order to improve the understanding of resource allocation in the mother plants in response to salinity, the seeds of individual fruits from each line and treatment were sorted according to maturity and counted (see Methods section). Only two lines, IL5-4 and IL7-4, had a significant difference in total seed number in SDS vs. SDF. The number of mature seeds per M82 fruit was significantly (*p* < 0.01) reduced, by nearly 50%, in SDS compared to SDF (Fig. 1). M82 also had a significant reduction in maturation percent, which was calculated as the number of mature seeds out of the total seed number, in SDS compared to SDF. All but one IL displayed a lower maturation percentage in SDS than in SDF, 13 of which were significant. Therefore, all lines were bulked for statistical analysis of the salinity effect in order to improve statistical power (Fig. 1). The bulked analysis confirmed that the decrease in the number of mature seeds and the increased number of aborted seeds per fruit was a significant (*p* < 0.0001) response to salinity displayed across the population. The numbers of aborted seeds per fruit in M82 was higher than the population average in both growth conditions. The number of mature seeds per fruit in M82 was slightly higher than the population average in SDF, but substantially lower than the average in SDS, reflected in a greater drop in maturation percentage for the control line. In contrast, the seed weight of M82 was close to the average weight of the ILs, and the total variation (presented by the error bars in Fig. 1) was small relative to the variation in both mature and aborted seed numbers.

Among SDF, 28 ILs had significant (*p* < 0.01) differences in maturation percentage compared to M82 (Additional File 1: Table S3), all of them showing increased maturation percentages ranging from 1.12- to 1.17-fold over M82. In SDS, all but one (IL8-1-1, which had a 0.9-fold decrease) of the 16 significantly differing ILs had increased (1.13–1.64 fold-change) maturation percent compared to M82. The ILs with a significant increase of maturation percent in both SDS and SDF (IL2-6, IL2-6-5, IL5-1, IL6-4, IL10-1-1, IL10-2 and IL11-4-1) likely indicate potential QTLs for genetic regulation of this trait. All lines with significant differences between SDS and SDF displayed an increase in aborted seed number, a decrease in mature seeds and, therefore, a reduction in maturation percent.

Following these findings, we hypothesize that within fruit competition, particularly under salinity stress, leads to abortion of part of the potential seeds to enable the remaining ones to develop to full maturity in order to maintain the typical, genetically determined, average seed weight to preserve seed quality when resources are limited. To elucidate whether the conservation of seed weight is accompanied by preservation of seed quality, the metabolite profile was evaluated by GC-MS, and germination trials were conducted.

### Metabolic effects of salinity

The relative content of soluble primary metabolites in the whole mature dry seed was determined by GC-MS. In samples from the whole population, 65 metabolites were annotated and quantified. Only annotated metabolites were included in the analysis. Among the metabolites that were annotated were amino acids, sugars, nitrogen containing metabolites, TCA cycle intermediates, organic acids and others that do not fall into these categories.

The difference in relative metabolite content (RMC) in the dry seed between SDS and SDF of each line was examined by a *t*-test for each line for the first season. Overall, 53 significant (*p* < 0.01) differences in RMC between SDS and SDF were detected out of 5153 pairwise comparisons (Table 2). The low number of significant changes may be due to the variability between replicate experimental plots, or due to an inherent resilience of seed metabolism to environmental factors associated with robust seed features, i.e., seed weight. No metabolite class had a distinct representation in the differences detected. Fructose, 2-hydroxyglutarate and dopamine each had significant changes in three ILs, while other metabolites differed significantly in one or two lines.

**Table 2:** Metabolites significantly differing in SDS compared to SDF

		RMC		RMC	
Line	Metabolite	SDF	(se)	SDS	(se)
IL3-2	Lactate	1.263	(0.17)	0.444	(0.04)^*^
	Isoleucine	1.123	(0.05)	0.683	(0.03)^**^
	Valine	1.384	(0.25)	0.439	(0.06)^*^
	Methionine	1.321	(0.21)	0.265	(0.06)^*^
	Aspartate	0.522	(0.07)	1.804	(0.1)^**^
	2OH glutarate	2.728	(0.78)	0.510	(0.12)^*^
	4OH benzoate	1.265	(0.17)	0.476	(0.06)^*^
	Arginine	1.646	(0.44)	0.417	(0.02)^*^
	Fructose	0.837	(0.11)	0.343	(0.03)^*^
	Lysine	0.976	(0.17)	0.343	(0.05)^*^
M82	MMP	1.305	(0.18)	0.632	(0.1)^*^
	Methionine	0.817	(0.12)	1.257	(0.12)^*^
	GABA	0.842	(0.09)	1.292	(0.08)^***^
	Cysteine	0.850	(0.08)	1.294	(0.1)^**^
	Asparagine	0.863	(0.08)	1.143	(0.07)^**^
	Ferulate	0.767	(0.06)	1.429	(0.15)^***^
IL2-1	Nicotinate	0.642	(0.06)	1.206	(0.11)^*^
	GABA	1.017	(0.21)	0.267	(0.07)^*^
	Alloinositol	0.730	(0.06)	1.149	(0.07)^*^
	Dopamine	0.570	(0.1)	1.898	(0.15)^*^
IL2-4	Glycerate	0.715	(0.2)	2.520	(0.15)^*^
	Fumarate	0.854	(0.16)	3.347	(0.45)^*^
	Alloinositol	1.767	(0.24)	0.528	(0)^*^
IL12-3	2OH glutarate	0.611	(0.06)	1.192	(0.09)^**^
	Fructose	0.472	(0.14)	1.953	(0.17)^*^
	Glucose	0.567	(0.09)	1.780	(0.18)^**^
IL3-1	Succinate	0.520	(0.07)	1.143	(0.12)^*^
	2OH glutarate	0.534	(0.05)	1.598	(0.25)^*^
IL7-1	Glycerol	0.355	(0.07)	0.968	(0.25)^*^
	Saccharate	1.263	(0.13)	0.579	(0.18)^*^
IL7-5	Tartarate	0.730	(0.16)	1.777	(0.24)^*^
	Saccharate	1.378	(0.07)	0.655	(0.15)^*^
IL1-1	Citrate	0.184	(0.03)	1.145	(0.2)^*^
IL1-1-3	Isoleucine	1.190	(0.08)	0.707	(0.07)^*^
IL1-3	Gluconate	1.082	(0.11)	1.864	(0.1)^*^
IL1-4	Glycerol 2 phosphate	0.564	(0.03)	0.873	(0.06)^*^
IL2-3	Tryptamine	2.284	(0.3)	1.072	(0.06)^*^
IL3-4	Aspartate	0.686	(0.09)	1.767	(0.27)^*^
IL3-5	Tyramine	0.965	(0.19)	0.189	(0.24)^*^
IL4-1-1	Arabinose	0.471	(0.04)	1.151	(0.18)^*^
IL4-2	Dopamine	0.879	(0.2)	0.199	(0.33)^*^
IL4-3-2	Galactarate	0.559	(0.04)	1.022	(0.58)^*^
IL5-3	Cysteine	2.114	(0.12)	1.092	(0)^*^
IL6-4	Arabinose	0.307	(0.06)	0.774	(0.42)^*^
IL7-3	Dopamine	3.641	(1.01)	0.636	(0.93)^*^
IL7-4-1	Sorbitol	0.231	(0.03)	1.143	(0.12)^**^
IL9-1	Alanine	0.476	(0.06)	0.935	(0.44)^*^
IL9-3	Glucose	2.714	(0.03)	0.628	(0.35)^*^
IL9-3-1	Arginine	1.832	(0.26)	0.864	(0.13)^*^
IL11-1	Fructose	0.277	(0.02)	0.497	(0.03)^**^
IL11-4-1	Glycerol	1.432	(0.17)	0.475	(0.09)^**^
IL12-2	Alanine	0.415	(0.05)	0.875	(0.05)^*^
IL12-4	Lactate	0.484	(0.05)	1.315	(0.19)^*^

The average RMC and standard error (se) in SDS and SDF for lines in which there was a significant difference between treatments

Asterisks mark significant differences between treatments of ^*^ *p* < 0.01, ^**^ *p* < 0.001, ^***^ *p* < 0.0001

Increased abundance in response to salinity treatment in M82 was observed for asparagine, cysteine, ferulate, γ-amino butyric acid (GABA) and methionine, but monomethylphosphate (MMP) had a 2-fold decrease. IL3-2 had the highest number (10) of significant (*p* < 0.01) changes in metabolite content of SDS compared to SDF. In this IL, aspartate increased and nine other metabolites (4-hydroxybenzoate, fructose, 2-hydroxyglutarate, Isoleucine, lactate, methionine, arginine, lysine and valine) decreased in SDS. IL2-1 had a decrease in GABA and an increase in nicotinate, alloinositol and dopamine. IL12-3 and IL2-4 displayed three instances of significantly changed metabolite abundance between treatments. Other ILs had two or fewer significant differences.

### Seed putative metabolic QTLs in SDF (f-QTLs), SDS (s-QTLs) and fold-change (FC)-QTLs

Potential loci for metabolite regulation were examined by comparing the RMC of each IL to M82 of seeds from plants grown under the same conditions. In SDF, 94 significant (p < 0.05; Bc: Bonferroni correction) differences were found between the ILs and M82. Chromosomes 1, 2 and 9 stand out in having many putative QTLs (Additional File 1: Table S4). The ILs showing the most abundant metabolite changes in comparison to M82 were: IL1-2 (11 f-QTLs), IL2-1-1 (8 f-QTLs) and IL9-2 (10 f-QTLs). The results for SDS (Additional File 1: Table S5) suggest 99 putative QTLs (p < 0.05, Bc). The most distinct ILs with many putative QTLs in the salinity treatment were IL2-1 (8 s-QTLs), IL2-1-1 (7 s-QTLs), IL2-1-1 also had a notable number of QTLs in SDF, IL3-2 (7 s-QTLs), IL3-4 (11 s-QTLs), and IL8-1-3 (8 s-QTLs).

In both SDS and SDF, a conserved relation between co-located putative QTLs of amino acids and other nitrogen compounds was found (Additional File 1: Tables S4-S5). For example, when most protein amino acids increase in abundance, GABA and ornithine increase as well, but urea and dopamine display a reduction, and vice versa. It is noteworthy that simple sugars (glucose, fructose, arabinose and sorbitol-sugar alcohol) frequently have joint QTLs, indicating putative loci of a shared regulation mechanism. In the present experiment, sucrose did not share QTLs with simple sugars, contrary to the findings by Toubiana et al. [25], which might suggest an environmental contribution to the sucrose level in seeds. In general, there was little overlap in specific putative QTLs between the separate treatment maps (Additional File 1: Tables S4-S5). However, chromosomes 1, 2 and 9, having several QTLs in both SDS and SDF maps, appeared to be important regulatory regions for seed metabolism (e.g., IL2-1-1). The strength of the IL2-1-1 QTL is enforced by matching s-QTLs for proline and methionine found in IL2-1 whose introgression segment contains that of IL2-1-1.

**Fig. 2:**
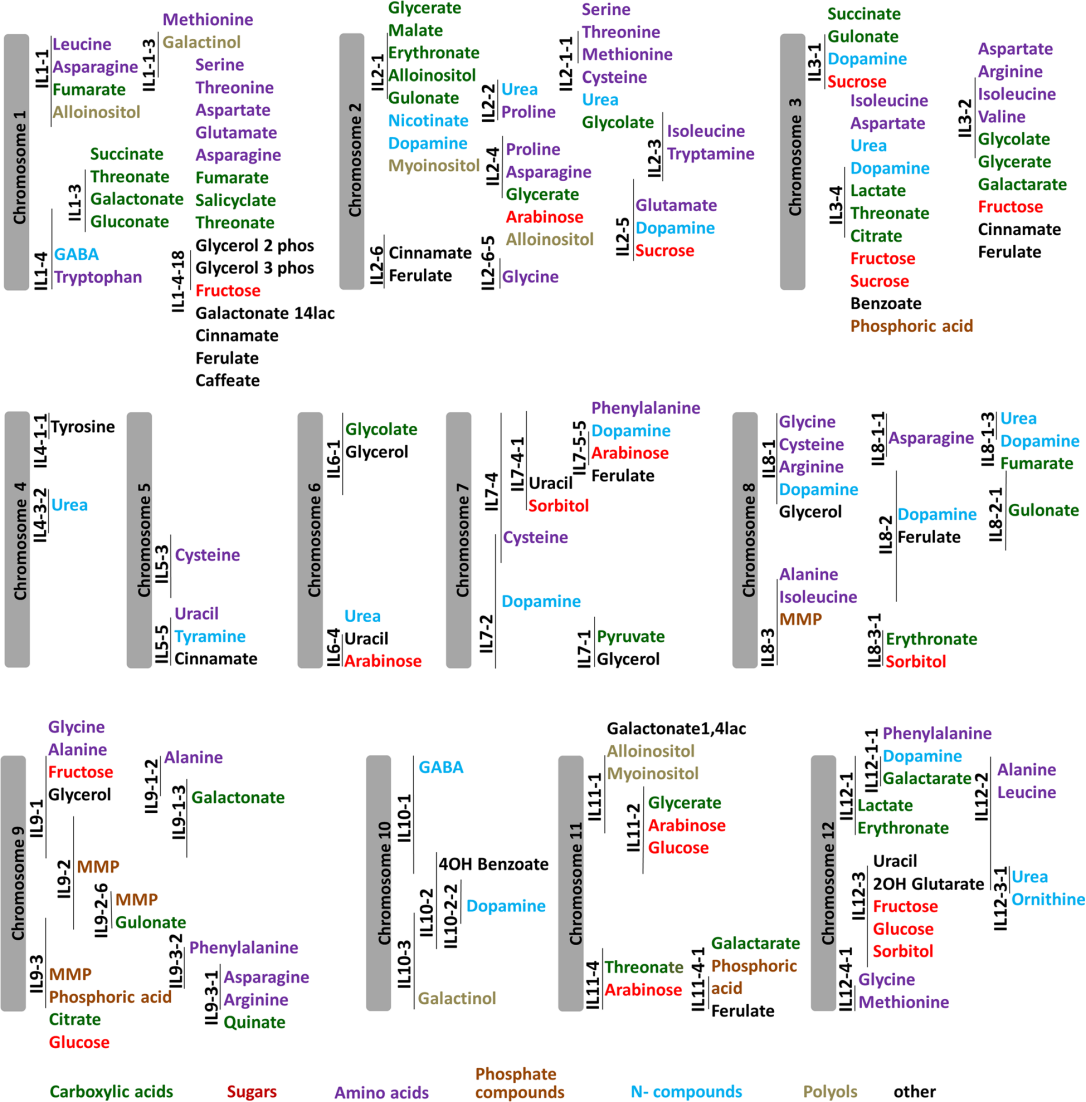
QTL map of metabolic response to salinity. Metabolites which had significant (*p* < 0.05, Bc) differences in FC in an IL compared to M82 are noted in parallel to the genomic location of the introgression segment. Colors represent metabolite class, as indicated

In order to locate the putative QTLs controlling the response to seed development under salinity, the FC of the RMC in SDS over SDF of each metabolite in each IL was compared to the respective FC of M82. A total of 167 putative QTLs for the metabolic response to salinity were detected (Fig. 2). The metabolites with the highest number of FC-QTLs were dopamine (11), urea (7), fructose (5) and ferulate (6). The ILs showing the highest number of significant (*p* < 0.05, Bc) differences in metabolite FC, compared to the M82 FC, were IL1-4-18 (15 FC-QTL), IL2-1 (8 FC-QTL), IL3-2 (10 FC-QTL) and IL3-4 (11 FC-QTL). The many FC-QTL and s-QTLs in IL3-2 and IL3-4 stand out in opposition to the few QTLs in SDF. The repeated metabolic alteration measurements in response to salinity in these lines suggest a noteworthy stress response element harbored in their introgression segments, which influences seed metabolism.

### Germination

**Fig. 3:**
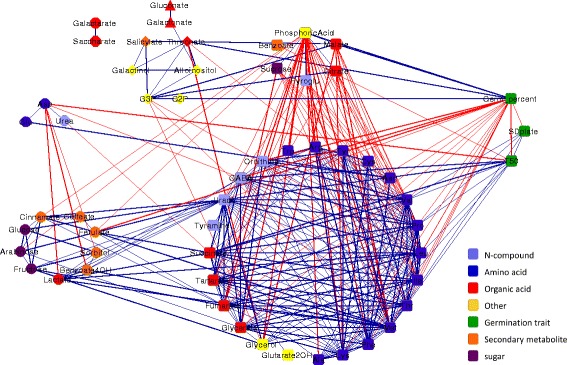
Correlation network of metabolites and germination traits of SDF. Nodes depict metabolites and germination measures. Metabolite nodes were arranged according to Walktrap communities (also indicated by node shape), and colored by chemical class, as indicated. Edges represent significant (*p* < 0.05, FDR; |*r*| > 0.4) correlations. Each community was separated according to positive (*blue*) and negative (*red*) correlations

**Fig. 4:**
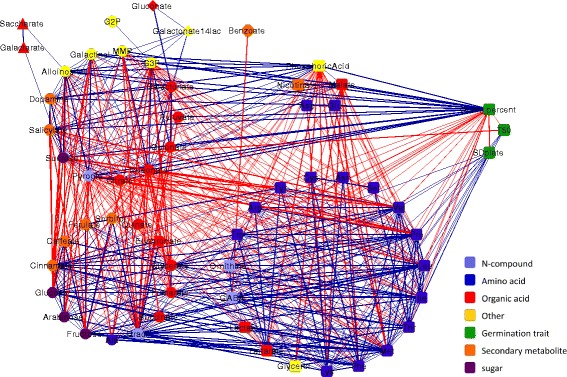
Correlation network of metabolites and germination traits of SDS. Nodes depict metabolites and germination measures. Metabolite nodes were arranged according to Walktrap communities (also indicated by *node shape*), and *colored* by chemical class, as indicated. Edges represent significant (*p* < 0.05, FDR; |*r*| > 0.4) correlations. Each community was separated according to positive (*blue*) and negative (*red*) correlations

In order to investigate the implication of the seed metabolite content and developmental conditions on germination vigor, three measures were quantified: (i) final germination percent, (ii) day of 50% germination (T50), as a measure of germination rate, and (iii) standard deviation of germination day within an experimental plate (SD-plate) as a measure of germination uniformity. There was a general correspondence and a significant (*p* < 0.0001) correlation between the three measures (Figs. 3 and 4). Germination percent was negatively correlated to T50 (*r* = −0.47, SDF; *r* = −0.51, SDS) and SD-plate (*r* = −0.54, SDF; *r* = −0.56, SDS). This shows that plots (each field plot consisting of four plants from which seeds were pooled) with a high germination percent tended to have a lower T50 and, thus, a faster germination rate and a low SD within the plates, resulting in more uniform germination. Plots displaying such traits would be considered to have high seed vigor. T50 and SD-plate were found to be positively correlated to each other (*r* = 0.59, SDF; *r* = 0.65, SDS). Despite the general correspondence, differences between the germination traits in QTLs and response to growth conditions were found.

Most ILs displayed no significant differences in germination in SDS compared to SDF. M82 and IL6-1 had a significant (*p* < 0.05) increase in T50 in SDS compared to SDF, and IL2-5 and IL11-4 had significant decreases in germination percent (Table 3), thus reflecting the reduced vigor of SDS. IL1-1-3 showed a decrease in T50, as well as an increase in germination percent. Additionally, IL2-1-1, IL3-4 and IL8-3-1 had a significantly increased germination percent in SDS, indicating improved seed vigor. IL4-1, IL7-4 and IL12-1 all had reduced SD-plate in SDS. It is noteworthy that despite the statistically significant differences, the extent of change in germination percent was mild and ranged from a 7% decrease to an increase of 14% in SDS compared to SDF. The impact of salinity on T50 and SD-plate was more severe, with two-day changes in T50 and reductions to 48-58% in SD within the plate, in the lines with significant differences.

**Table 3:** Germination response to salinity

	FC %	FC T50	FC SDplate
M82	0.954	1.286^*^	1.815
IL1-1-3	1.186^*^	0.615^**^	0.632
IL2-1-1	1.021^*^	0.833	0.655
IL2-5	0.931^*^	1.000	0.936
IL3-4	1.155^*^	1.000	1.392
IL4-1	1.037	1.000	0.579^**^
IL6-1	0.724	1.500^*^	1.349
IL7-4	1.318	0.517	0.584^*^
IL8-3-1	1.029^*^	1.000	1.749
IL11-4	0.967^*^	0.857	1.750
IL12-1	1.010	0.750	0.486^*^

The ratio of germination measurements in SDS to SDF for lines in which there was a significant difference between treatments

Asterisks mark significant differences between treatments with the Bcp of ^*^ *p* < 0.05, ^**^ *p* < 0.01, ^***^ *p* < 0.001

A higher number of significant differences in germination were seen in the comparison of the ILs to M82 (Table 4). In SDF, three ILs (IL1-1-3, IL3-4 and IL9-2-6) had a significantly lower germination percent compared to M82 and eight ILs (IL2-1-1, IL2-6, IL6-4, IL7-1, IL7-5-5, IL8-1-3, IL10-2 and IL11-4) had higher. In SDS, all eleven ILs (IL2-1-1, IL3-2, IL3-4, IL5-1, IL7-3, IL7-4, IL7-5-5, IL8-3-1, IL9-2-5, IL9-3-1 and IL12-1) with significant differences displayed a higher germination percent than M82. IL2-1-1, IL3-4 and IL7-5-5 had significant advantages over M82 under both conditions.

**Table 4:** Putative germination QTLs for SDF and SDS

	SDF						SDS					
	%	(se)	T50	(se)	SDplate	(se)	%	(se)	T50	(se)	SDplate	(se)
M82	95.13	(0.89)	2.66	(0.14)	1.00	(0.08)	90.75	(2.75)	3.42	(0.32)	1.82	(0.76)
IL1-1-2	97.33	(1.33)	2.33	(0.33)	0.87	(0.24)	95.94	(2.06)	3.00	(0)	0.92	(0.13)
IL1-1-3	79.24	(4.20)^*^	5.00	(0.40)^***^	1.49	(0.22)	93.94	(1.50)	3.08	(0.07)	0.94	(0.13)
IL1-3	87.03	(6.78)	3.00	(0.57)	0.96	(0.14)	76.75	(16.5)	3.83	(0.44)	1.10	(0.45)
IL1-4	76.04	(23.9)	4.00	(1)	1.63	(0.74)	77.00	(23)	4.25	(1.75)	1.56	(0.74)
IL1-4-18	95.99	(1.14)	2.50	(0.28)	1.08	(0.13)	93.78	(2.32)	2.00	(0)^***^	0.73	(0.22)
IL2-1	91.16	(1.73)	3.00	(0)^**^	0.73	(0.07)	97.33	(0.66)	2.33	(0.33)	0.47	(0.00)^***^
IL2-1-1	97.91	(0.08)^**^	3.00	(0)^**^	1.41	(0.13)^*^	100.00	(0)^*^	2.50	(0.5)	0.93	(0.10)
IL2-2	76.52	(10.3)	3.50	(0.56)	1.35	(0.30)	96.00	(1.54)	3.17	(0.30)	0.98	(0.05)
IL2-3	91.33	(1.33)	3.33	(0.33)	1.27	(0.25)	67.74	(NA)	4.00	(NA)	1.62	(NA)
IL2-5	98.67	(1.33)	3.00	(0.57)	1.14	(0.32)	91.87	(1.04)	3.00	(0)	1.07	(0.08)
IL2-6	98.00	(0)^**^	3.00	(0)^**^	0.65	(0.11)	91.00	(1)	3.00	(0)	1.50	(0.18)
IL2-6-5	97.21	(1.83)	3.00	(0)^**^	0.84	(0.22)	97.96	(2.04)	3.00	(0)	1.01	(0.38)
IL3-1	94.00	(6)	2.50	(0.5)	1.44	(0.17)	90.67	(6.56)	3.67	(0.33)	1.05	(0.24)
IL3-2	86.65	(4.40)	3.38	(0.37)	1.28	(0.49)	98.50	(0.95)^*^	2.00	(0.40)	0.89	(0.16)
IL3-4	85.71	(0.29)^***^	4.00	(1)	0.85	(0.29)	99.00	(1)^*^	4.00	(0)^**^	1.18	(0.37)
IL3-5	95.97	(1.98)	3.00	(0.57)	1.72	(0.21)^*^	96.67	(3.33)	3.00	(0.57)	0.87	(0.36)
IL4-1	92.58	(3.29)	3.00	(0)^**^	2.21	(0.16)^***^	95.98	(1.97)	3.00	(0)	1.28	(0.06)
IL4-1-1	97.33	(2.66)	3.00	(0)^**^	0.89	(0.12)	92.00	(4)	3.50	(0.5)	1.29	(0.38)
IL4-2	98.00	(1.15)	3.00	(0.57)	1.21	(0.36)	49.83	(8.16)	8.25	(2.25)	2.40	(0.22)^***^
IL4-3	92.67	(7.33)	4.67	(1.20)	1.53	(0.63)	95.00	(5)	3.50	(0.5)	1.21	(0.00)
IL4-3-2	93.93	(2.36)	3.33	(0.33)	1.19	(0.34)	90.67	(6.56)	4.33	(0.33)^*^	1.32	(0.53)
IL5-1	100.00	(NA)	2.00	(NA)	0.86	(NA)	99.00	(1)^*^	4.00	(0)^**^	1.26	(0.19)
IL5-3	92.67	(6.35)	4.00	(1)	1.89	(0.61)	91.00	(1)	3.50	(0.5)	1.11	(0.20)
IL5-4	97.00	(1)	3.25	(0.25)	1.23	(0.18)	98.00	(NA)	4.00	(NA)	1.44	(NA)
IL5-5	97.00	(1)	3.00	(0)^**^	0.43	(0.11)	92.00	(4)	3.33	(0.66)	1.18	(0.05)
IL6-1	80.00	(8.32)	3.33	(0.33)	1.94	(0.22)^**^	57.92	(19.6)	5.00	(0)^***^	2.62	(0.07)^***^
IL6-4	100.00	(0)^***^	1.68	(0.16)^*^	1.26	(0.79)	95.33	(2.90)	2.87	(1.10)	0.89	(0.25)
IL7-1	99.33	(0.66)^**^	2.33	(0.33)	0.65	(0.02)^**^	100.00	(NA)	2.00	(NA)	0.75	(NA)
IL7-2	96.67	(2.40)	2.00	(0)^***^	1.02	(0.12)	94.04	(3.96)	2.00	(0)^***^	0.94	(0.35)
IL7-3	82.83	(12.8)	3.00	(1)	1.23	(0.44)	100.00	(0)^*^	2.50	(0.5)	0.23	(0.22)
IL7-4	75.10	(8.48)	4.83	(1.16)	1.89	(0.05)^***^	99.00	(1)^*^	2.50	(0.5)	1.10	(0.05)
IL7-4-1	98.00	(1.15)	3.00	(0.57)	0.76	(0.14)	96.67	(3.33)	3.33	(0.66)	1.49	(0.02)^*^
IL7-5	94.67	(2.40)	2.33	(0.33)	1.32	(0.23)	95.92	(4.08)	4.00	(1)	1.68	(1.06)
IL7-5-5	99.00	(1)^*^	3.00	(0)^**^	1.22	(0.33)	98.00	(1.15)^*^	2.67	(0.33)	0.82	(0.16)
IL8-1	96.63	(0.66)	2.33	(0.33)	0.59	(0.13)	90.47	(7.49)	3.00	(0)	1.02	(0.24)
IL8-1-1	98.00	(2)	2.00	(0)^***^	0.39	(0.04)^*^	92.00	(NA)	3.00	(NA)	2.05	(NA)
IL8-1-3	99.33	(0.66)^**^	3.67	(0.33)^*^	1.39	(0.03)^***^	97.00	(1)	3.00	(0)	0.98	(0.21)
IL8-2	92.00	(3.05)	3.67	(0.33)^*^	1.52	(0.18)^*^	96.67	(0.66)	3.00	(0)	0.93	(0.41)
IL8-2-1	96.00	(4)	3.00	(0)^**^	1.00	(0.18)	95.16	(0.71)	2.67	(0.33)	1.24	(0.07)
IL8-3	94.44	(5.55)	3.00	(0.57)	1.04	(0.42)	97.92	(2.08)	3.00	(0)	1.23	(0.72)
IL8-3-1	97.22	(0.61)	2.33	(0.33)	0.58	(0.13)	100.00	(0)^*^	2.33	(0.33)	1.01	(0.23)
IL9-1	95.57	(1.59)	2.40	(0.24)	0.98	(0.22)	75.00	(8.37)	3.40	(0.48)	1.65	(0.30)
IL9-1-2	95.00	(3)	3.00	(0)^**^	1.49	(0.21)	94.67	(2.90)	3.33	(0.66)	1.73	(0.65)
IL9-1-3	69.12	(6.41)	3.13	(0.24)	1.29	(0.15)	74.56	(13.8)	3.30	(1.15)	1.65	(0.35)
IL9-2	82.67	(11.0)	3.00	(0)^**^	1.14	(0.19)	93.00	(3)	3.00	(0)	0.64	(0.10)
IL9-2-5	96.00	(2.30)	3.05	(0.62)	0.84	(0.16)	99.00	(1)^*^	2.00	(0)^***^	0.43	(0.16)
IL9-2-6	89.90	(0.10)^***^	5.50	(0.5)^*^	1.90	(0.29)	74.00	(22)	5.17	(0.44)^**^	1.83	(0.39)
IL9-3	71.54	(13.3)	4.67	(1.45)	1.64	(0.23)^*^	78.72	(12.9)	4.00	(1.52)	1.39	(0.54)
IL9-3-1	98.00	(2)	2.75	(0.25)	0.81	(0.03)	100.00	(0)^*^	3.00	(0)	0.98	(0.53)
IL9-3-2	97.33	(2.66)	3.33	(0.33)	1.71	(0.26)^*^	95.16	(1.82)	3.67	(0.33)	1.85	(0.35)
IL10-1	92.67	(6.35)	3.67	(0.33)^*^	1.19	(0.30)	94.00	(NA)	3.00	(NA)	1.19	(NA)
IL10-1-1	85.33	(4.37)	4.00	(0.57)	1.70	(0.12)^***^	81.33	(6.35)	4.67	(0.88)	2.02	(0.14)^**^
IL10-2	100.00	(0)^***^	2.50	(0.5)	0.93	(0.18)	100.00	(NA)	3.00	(NA)	0.81	(NA)
IL10-2-2	93.33	(6.66)	3.00	(1)	0.92	(0.27)	91.17	(7.85)	3.67	(0.66)	1.19	(0.29)
IL10-3	77.35	(9.03)	3.33	(0.88)	1.91	(0.50)	77.71	(6.28)	5.00	(1)	2.35	(0.67)
IL11-2	90.00	(4)	4.50	(0.5)	1.04	(0.29)	96.67	(3.33)	4.00	(1)	1.45	(0.86)
IL11-3	98.67	(1.33)	4.67	(0.33)^**^	1.66	(0.09)^***^	88.64	(8.34)	5.00	(0)^***^	1.73	(0.06)^**^
IL11-4	100.00	(0)^***^	3.50	(0.5)	0.68	(0.09)	96.67	(0.66)	3.00	(0)	1.19	(0.42)
IL11-4-1	92.00	(6)	4.00	(0)^***^	1.32	(0.34)	92.67	(6.35)	4.00	(0.57)	1.35	(0.36)
IL12-1	98.00	(2)	4.00	(1)	1.55	(0.06)^***^	99.00	(1)^*^	3.00	(0)	0.75	(0.00)
IL12-1-1	96.96	(0.95)	3.00	(0)^**^	0.79	(0.23)	90.14	(6.79)	3.00	(0.57)	0.99	(0.11)
IL12-2	91.96	(2.93)	2.77	(0.32)	0.82	(0.10)	88.80	(4.14)	2.44	(0.39)	0.83	(0.14)
IL12-3	89.18	(6.49)	3.67	(0.33)^*^	1.14	(0.10)	92.00	(NA)	3.00	(NA)	1.01	(NA)
IL12-3-1	96.00	(4)	3.50	(0.5)	1.22	(0.30)	97.92	(2.08)	4.00	(1)	1.18	(0.33)
IL12-4	76.67	(16.3)	4.17	(1.16)	1.80	(0.70)	92.00	(6)	2.50	(0.5)	1.14	(0.13)
IL12-4-1	95.83	(4.16)	3.00	(1)	1.10	(0.44)	94.00	(2)	2.50	(0.5)	0.94	(0.11)

Germination measurements of M82 and ILs with significant differences compared to M82 within the same treatment

Asterisks mark significance levels with the Bcp of ^*^ *p* < 0.05, ^**^ *p* < 0.01, ^***^ *p* < 0.001

Only three ILs (IL6-4, IL7-2 and IL8-1-1) were found to have significantly lower T50 than M82, and 20 ILs (IL1-1-3, IL11-4-1, IL2-1, IL2-1-1, IL2-6, IL2-6-5, IL4-1, IL4-1-1, IL5-5, IL7-5-5, IL8-1-3, IL8-2, IL8-2-1, IL9-1-2, IL9-2, IL9-2-6, IL10-1, IL11-3, IL12-1-1 and IL12-3) had T50 higher than M82 for SDF. Two of these ILs, IL11-3 and IL9-2-6, also had higher levels than M82 in SDS. Additional ILs with elevated T50, compared to M82 in SDS, were IL3-4, IL4-3-2, IL5-1 and IL6-1. Faster germination, i.e., lower T50, was found in IL1-4-18, IL7-2 (also in SDF) and IL9-2-5 in SDS. IL1-1-3 had a slower and lower germination percent than M82 in SDF but significantly improved in response to salinity in SDS, leading to a level similar to M82 in SDF.

For SDF, most differing ILs (12 in number; IL2-1-1, IL3-5, IL4-1, IL6-1, IL7-4, IL8-1-3, IL8-2, IL9-3, IL9-3-2, IL10-1-1, IL11-3 and IL12-1) had a higher SD-plate than M82. IL7-1 and IL8-1-1 had a lower SD-plate in SDF. IL6-1 and IL10-1-1 and IL4-2 had a higher SD-plate in SDS. Three ILs had a decrease in SD-plate in SDS: IL2-1, IL7-4-1 and IL11-3. Interestingly, IL11-3 had a higher than M82 SD-plate in SDF and a lower one in SDS, but had a consistently higher T50 in both conditions.

It is noteworthy that IL2-1-1 was the only IL that had a significantly higher germination percent than M82 for both SDS and SDF, and a significantly elevated germination percent for SDS. However, in SDF, both T50 and SD-plate were also higher. Notably, a high number of significant metabolite differences also characterized this IL compared to M82 in both conditions (a map with QTLs overlapping in germination and metabolite traits in SDS can be found in Additional File 2). IL3-4 had a lower germination percent than M82 in SDF but had a significant increase in SDS, leading to an improved germination percent over M82 in SDS, though at a slower rate. This same IL had a high number of significant metabolic changes in response to salinity. These results emphasize the correspondence existing between genetic and metabolic factors modulating germination and their interaction with the maternal environment.

Taken together, the analysis of the IL population revealed putative genomic regions (i.e., QTLs) regulating the measured seed traits. Comparison of the QTL locations for the various traits and conditions revealed co-localization of metabolic changes and of changes in germination traits. Subsequently, multivariate analysis methods were applied to study the variation and correlations between traits in the population.

### Population-wide integrative analysis

Previous studies have shown the potential for using the intrinsic variation in metabolic content within a population for inferring biologically significant correlations [25, 36]. Here we used a correlation network analysis to investigate the relations between metabolite co-response to genetic alteration and to the maternal environment and the seed germination.

### Network analysis revealed a conserved network topology under various conditions

In order to examine the inter-relations between the metabolite changes, pairwise correlations of RMC of all metabolite pairs were calculated for each of the field conditions. Correlation results from the first season were used to construct a network for visualization and for graph-based analysis (Figs. 3 and 4). Metabolite correlations from the second season showed the strongly correlated groups of metabolites preserved while the networks had lower connectivity (Additional Files 3 and 4). The significance thresholds (*p* < 0.05; false discovery rate (FDR): |*r*| > 0.4) were optimized using graph theory measures as performed by Fukushima et al. [37]. Significant correlations were depicted as edges between the nodes, representing metabolites and germination traits. Only metabolites that had significant correlations were included in the networks. The network based on the SDS dataset included 527 significant correlations connecting 60 nodes (Table 5). The SDS network had a higher density than the SDF network, which had 301 edges and 55 nodes. The greater interconnectivity of the SDS network was reflected by a variety of measures, including a higher average nodal degree and clustering coefficient, and a lower average path length and network diameter. Networks based on stress conditions have previously been found to have higher connectivity than networks based on control conditions in grapevine [38]. The two networks, based on SDS and SDF, had more positive correlations than negative ones, a feature already noted in previous studies [25].

**Table 5:** Network Attributes

	SDF	SDS
Number of nodes	55	60
Number of positive edges	223	325
Number of negative edges	78	202
Total number of edges	301	527
Ration edges/nodes	5.472	8.783
Average nodal degree	8.852	15.5
Network diameter	7	4
Network density	0.132	0.231
Average path length	2.319	1.888
Clustering coefficient (transitivity)	0.607	0.643

Attributes of the correlation networks constructed from SDF and SDS seed metabolite levels

All metabolites were divided into communities using the “Walktrap” algorithm. Under both growth conditions, the metabolites formed two large communities with several additional small communities. We found that particularly conserved communities in the network tended to include metabolites sharing a biochemical pathway, indicating a stronger coordination of biochemically related metabolites, which supports other studies [37, 39].

The first and largest community included most amino acids, some organic acids and other metabolites. Aspartate and glutamate were usually correlated to each other and were found to be negatively correlated to the amino acids of the central cluster. Aspartate and glutamate are primary precursors for amino acid biosynthesis by transfer of an amino group to oxaloacetate and 2-oxoglutarate, respectively ([39]; KEGG). The negative correlations between the downstream amino acids and aspartate and glutamate is most likely due to their precursor-product relation in amino acid metabolism [40]. The positive correlations between the downstream amino acids, such as lysine, threonine and isoleucine for aspartate, and proline and arginine for glutamate, demonstrate their coordinated level of biosynthesis.

The second large community included the simple sugars and most secondary metabolites. The simple sugars (fructose, glucose and arabinose) were strongly correlated in our networks, though with more differences between seasons, as previously described [25]. For fructose and glucose, it is not surprising given their related metabolic pathway and utilization. Arabinose, however, is not known to share its metabolic pathways (KEGG, PlantCyc: [41]), but it was closely correlated to the hexoses in both growth conditions of the first season. The finding that sucrose is not immediately correlated to the simple sugars may be surprising, but has been seen previously [42]. Secondary metabolites of the phenylpropanoid biosynthesis pathway (KEGG; cinnamate, ferulate, caffeate) were consistently correlated and clustered in the second community. They were also positively correlated to their precursor phenylalanine, which was clustered in the central amino acid community. Phenylalanine products in other pathways, benzoate and salicylate, were negatively correlated to them. This is possibly a sign of competition between the pathways.

In the network based on SDF, a third community was formed of alloinositol, galactinol, salicylate, threonate, glycerol-3-phosphate and glycerol-2-phosphate. In the SDS network, these metabolites (excluding glycerol-2-phosphate) were interlinked with those of the second community and formed one community.

Each of the two large communities included metabolites with both positive and negative correlations. In order to better explore the landscape of relations among the metabolites, each community in the network was divided into subsets of metabolites positively correlated to each other and negatively correlated to the other subset (Figs. 3 and 4). The positive and negative correlations in the communities corresponded in a manner that created two overall metabolite sets. The first metabolite set 1 (ms1), shown on the lower half of the network graphs, included subsets of the two communities with positive correlations between all metabolites. The second metabolite set (ms2), shown on the upper half of the network graphs, included subsets of the two communities and additional small communities with all metabolites positively correlated to each other. The partition of metabolites into the two sets was consistent between the network graphs of growth conditions and seasons. Metabolite classes were represented in both sets. While consistent relations between metabolites within the same pathways are expected, the conserved nature of positive and negative relations between metabolites from seemingly unrelated pathways, such as aspartate, malate, dopamine and phosphoric acid, was not automatically expected. The consistency of metabolite partitioning hints at a fundamental metabolic balance with biological relevance, which we studied further in relation to germination.

### The seed metabolic profile is related to germination vigor

**Fig. 5:**
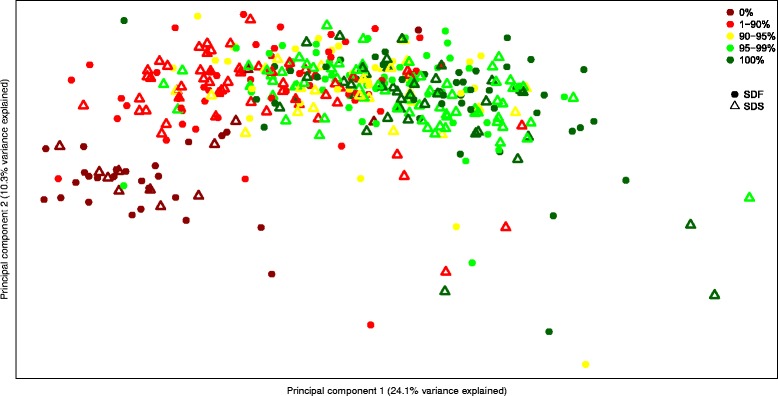
PCA of seed metabolic content with germination percent. PCA of metabolic content of the seed distinguishes between poorly and highly germinating seeds, but not between SDF and SDS. Dots are located on axis according to PCA of primary metabolite content. Full circles represent SDF and empty tringles represent SDS. Dots are colored by final germination percentage, as indicated

In order to assess the global effect of dry seed metabolite levels on germination, the metabolic profiles of all seed batches evaluated for germination were subjected to principal component analysis (PCA). SDF plots are marked with full circles and SDS plots are marked with empty triangles. Each data point was then colored according to the germination value associated with it (Fig. 5). There was no notable separation between SDF and SDS plots. With few exceptions, all samples that did not germinate were grouped together, and the rest of the samples were dispersed along the x axis (principal component 1; PC1) ranging from low germinating seed plots on the left to high germination plots on the right. The non-germinating phenotype may have resulted from post-harvest stress. The PCA and the false-scale coloring of the samples demonstrate a link between the seeds’ metabolic profile and their germination ability.

The metabolites that had the highest contribution to the variance in PC1 included methionine, proline, lysine, uracil, leucine and GABA. The second component (PC2) showed a separation between non-germinating and germinating seeds. PC1 and PC2 explained 28% and 10% of the variance, respectively. In PC2, glucose displayed the most negative eigenvalue, and dopamine, galactinol, threonate and tyramine had the most positive (i.e., higher in germinating seeds).

**Fig. 6:**
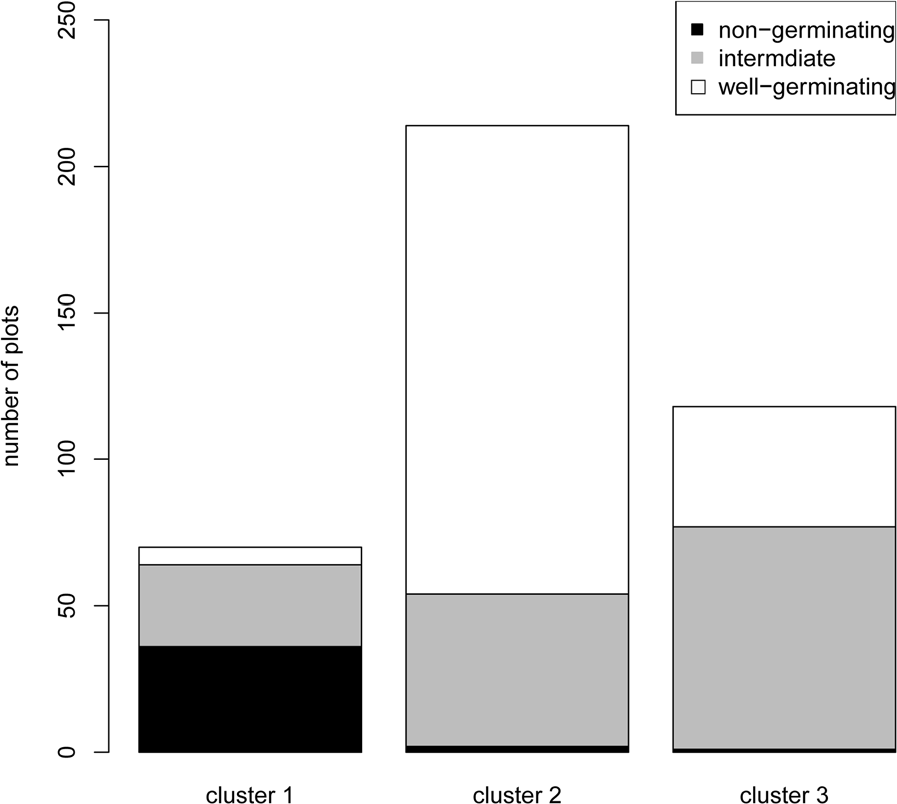
Distribution of germination groups among the K-means clusters. Plots tested for germination were clustered according to their RMCs using K-means clustering. The abundances of non-germinating, intermediate germination (5–95%) and well-germinating (95–100%) plots are indicated in *black*, *gray* and *white*, respectively

The K- means clustering (KMC) method (tMeV; [43]), based on RMC, was next applied to the dataset. The inclusion of non-germinating (0% germination), intermediate-germinating (1–94%) and well-germinating (95–100%) plots in each cluster was tested by chi-square. There was a significant (*p* < 0.0001) differential distribution of the germination groups between the three metabolite-based clusters (Fig. 6) compared to their distribution in all plots. Cluster 1 contained all but three of the non-germinating plots and was significantly enriched in this germination group (51.43% compared to 9.7% of all sampled plots). The cluster analysis validated the PCA and network analysis. For instance, metabolites that had low abundance in cluster 1 were from ms2 and were the same metabolite with the most positive eigenvalues in PC2, which separated non-germinating from germinating seeds. The second cluster was significantly dominated by well-germinating plots, which made up 74.77% of this cluster, compared to 51.49% of all sampled plots. The RMC pattern of this cluster generally opposed the patterns found in cluster 1. For example, the amino acids of the central module in ms1 were in low abundance and the metabolites of ms2 had high levels. The third cluster comprised 64.41% of the intermediate-germinating plots (compared to 38.81% of all sampled plots) and 34.75% well-germinating seeds. The metabolites with the lowest levels in this cluster were glucose, fructose, arabinose and urea. The central amino acids and nitrogen containing compounds of ms1 were found in high abundance in this cluster.

**Fig. 7:**
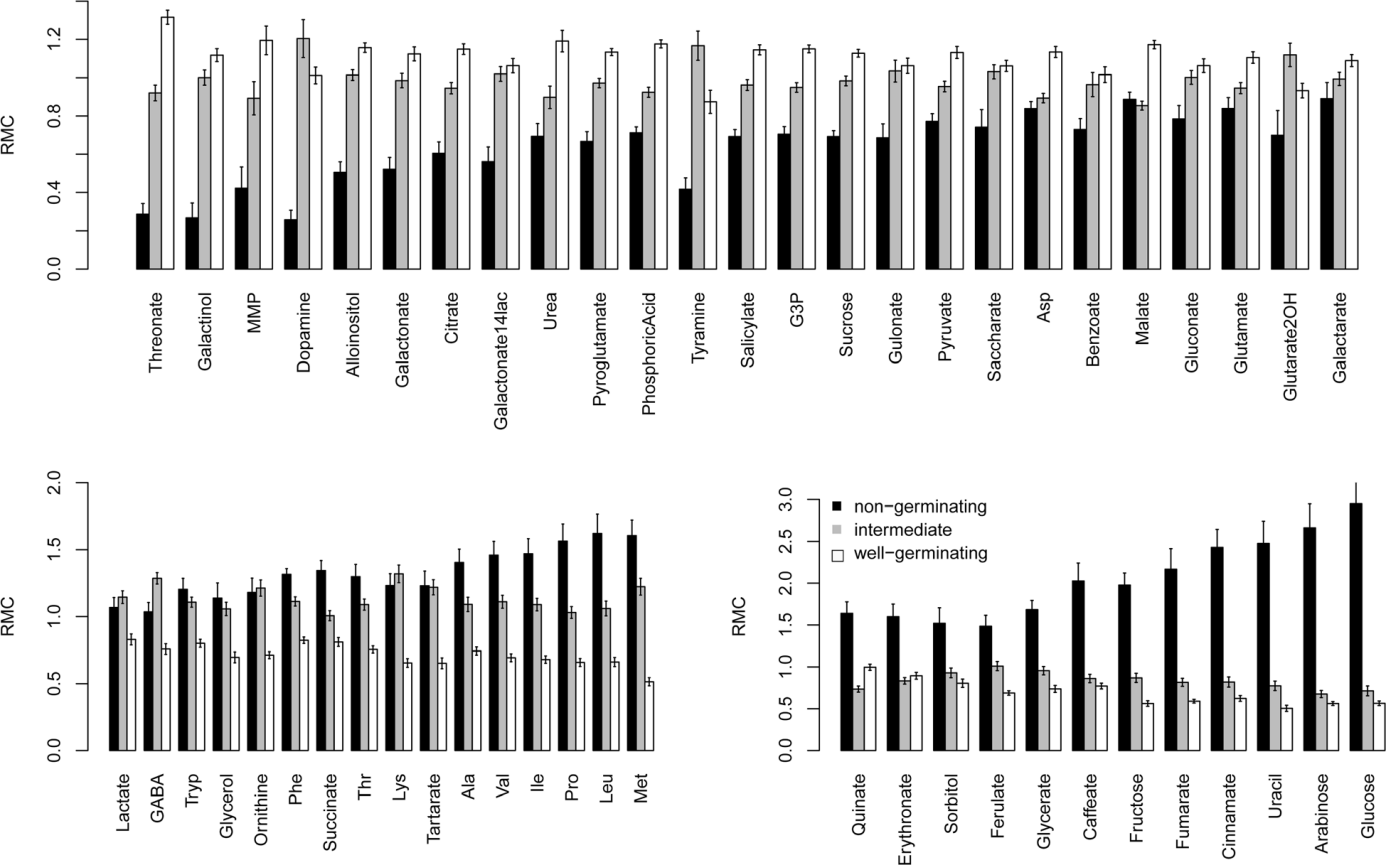
Seed RMC by germination groups. Plots tested for germination were divided according to level of germination into non-germinating (0% germination), intermediate germination (5–95%) and well-germinating (95–100%), indicated in *black*, *gray* and *white*, respectively. The RMC of each germination group is presented, for metabolites that significantly (*p* < 0.05, Bcp) differed in RMC between non-germinating and well-germinating plots. **a** Metabolites of ms2. **b** Metabolites of ms1 of the first community. **c** Metabolites of ms1 of the second community. *Error bars* represent standard error

T-tests were performed to identify the metabolites that were significantly (*p* < 0.05; Bcp: Bonferroni correction permissive) different between the well-germinating and non-germinating plots (Fig. 7), and the results confirmed the PCA, the KMC and the network communities grouping. Glucose showed a 5.2-fold significantly higher RMC in non-germinating plots than in well-germinating plots. The other metabolites from the second community of ms1 (Fig. 7c) also displayed high differences between non-germinating seeds and all levels of germinating seeds; for example, arabinose, uracil, cinnamate, fumarate, fructose and methionine had more than 3-fold higher RMC levels in non-germinating seeds. The amino acids and other metabolites from the first community of ms1 (Fig. 7b) displayed a different pattern in which plots with intermediate levels of germination had intermediate RMC levels. Metabolites of ms2, such as threonate, galactinol, MMP and dopamine, were significantly lower in non-germinating seeds. For this group, most metabolites had intermediate levels in intermediate-germinating seeds (Fig. 7a).

### Metabolite-germination correlations were consistent with the metabolite correlation sets

In order to study the relation between metabolites and germination, germination traits were integrated into the network analysis (Figs. 3 and 4). All the metabolites with negative correlations to germination percent or positive correlations to T50 or to SD plate were grouped in ms1 under both growth conditions. The metabolites with the strongest negative correlation to germination percent (*p* < 0.05 FDR, *r* < −0.4, ordered by r in both networks) were proline, methionine, leucine and lysine. All metabolites with positive correlations to germination percent and negative correlations to T50 were within ms2. Metabolites with the strongest positive correlation to germination percent (*p* < 0.05 FDR, *r* > 0.4, ordered by r in both networks) were threonate, phosphoric acid, malate and glycerol-3-phosphate. Sucrose was negatively correlated to T50 in the SDS network and to aspartate and benzoate in the SDF network. Amino acids and other metabolites from the first community in ms1 were positively correlated to T50 and SD-plate, in both networks. No metabolites had significant negative correlations to SD-plate, in either network.

Taken together, these results demonstrate a strong association between the balance of metabolite levels between ms1 and ms2 and the germination percent and rate. The four groups of metabolite communities and sets presented in the network topology represent four major patterns of association between RMC and germination. This grouping was reflected in the various types of analyses: PCA, KMC and RMC comparison in the germination percentage groups.

## Discussion

In the present study, we harnessed the natural variability of the introgression line population to explore the effects of genetics and maternal environment on seed traits and metabolism and the latter’s implications on germination. We identified putative genetic loci for tomato seed quality traits and metabolism, in response to different maternal environments. We further showed that the relative levels of metabolites in the dry seed can indicate the germination potential of the seed.

*S. pennelli* has much lighter seeds than the cultivated tomato M82 [35], and seed weight varied across the population. ILs displayed consistent differences in seed weight, demonstrating the same trend under different conditions (treatments and seasons). These results and the tight genetic control of the trait are in line with earlier works in intercrossed populations [44, 45]. The inverse relation between seed number and size has been long known both across species [46] and within the same species [45]. However, when resources are limited, a plant tends to reduce seed number while maintaining seed weight [47]. Seed weight may also be affected under severe stress [48, 49]. The environmentally induced abortion of seeds after seed set, as shown in our study, in place of producing fewer ovules, is a known strategy among plants [50] to regulate seed number for resource-expensive seed filling [51]. In our study, saline conditions affected seed number but not seed weight. Likewise, individual fruit weight was less affected than fruit number by the salinity stress. Still, overall fruit yield was negatively affected by salinity in all ILs, as was fresh vegetative weight in most ILs (Perelman, unpublished observations). The stability of seed weight in all lines, amid the environmental perturbation, points to its fundamental evolutionary advantage.

Several reports have suggested a positive relation between seed size, emergence [52, 53] and seeding vigor, especially under stress conditions [54]. Indeed, Khan et al. [44] found that seed weight in a tomato recombinant inbred line (RIL) population was correlated to seedling vigor measurements, but not to germination percentage, T50 and uniformity, which is similar to our findings. We hypothesized that maintaining seed weight leads to conservation of seed quality and germination vigor. This hypothesis was supported by the relatively low number of significant metabolic changes in the seeds and the overall moderate germination differences in response to salinity. Nevertheless, among those seeds that reached maturation, no clear relation was found between seed weight and either metabolic content or germination vigor traits, suggesting the occurrence of a threshold at which other determinants come into play.

The stability of multiple QTLs for seed weight under varying conditions suggests the important role of seed weight (and vigor) in plant evolution. Low levels of QTL co-localization for seed size, seed numbers and maturation percent were also observed (e.g., IL7-4-1, IL8-3-1 and IL11-4-1). The maturation percent of IL7-4-1 was reduced by salinity, but the maturation percent of the other ILs was not. Study of this introgression may lead to improved understanding of this trait’s regulation by the environment.

### Germination QTLs and the effect of maternal growth conditions

The germination of tomato seeds in saline conditions has been widely studied [27–29]. However, to our knowledge, no record is available on the germination of tomato seeds matured under different environmental or stress conditions. Trans-generational stress implications have been studied in other species. Lower germination was found for soybeans from a mother plant grown under conditions of severe drought and high temperature [48] and in leaf removal experiments in radish [55]. It has been shown in numerous studies that environmental conditions, such as temperature, light [56] and possibly others, during lettuce seed maturation have an effect on seed germination and plant growth. Lettuce seeds that developed at higher temperatures had reduced weight but germinated at higher percentages [57], specifically under higher than optimal germination temperatures [58, 59]. In Arabidopsis, temperature during development was found to have a strong influence on germination [33, 34], and higher light intensity, photoperiod, nitrate and phosphate also increased germination under stress conditions [32]. These studies demonstrate the positive and negative effects of maternal stress on offspring.

Despite the fact that *S. pennelli* is known as being more tolerant to stress than cultivated tomato variety M82 [28], QTLs conferring either higher or lower germination compared to M82 were found in both SDS and SDF. Other studies have found evidence of what could be considered transgressive segregation whereby alleles from the “tolerant” parent can contribute to an unexpected “sensitive” phenotype [24, 60–63]. A number of germination QTLs were found under both conditions, indicating independent genetic factors influencing germination, while other QTLs were detected only under one condition, suggesting environmentally responsive QTLs. Examples are present in the introgression segments IL1-1-3, IL2-1-1, IL3-4 and IL8-3-1, which had improved germination and IL2-5, IL6-1, IL11-4, which showed reduced germination in SDS compared to SDF. ILs that did not differ in germination, compared to M82, in SDF but did in SDS are potential candidates for harboring loci of maternal environmental response.

Consistency was observed between the QTLs for germination found in the present study and QTLs previously identified using an *S. esculentum* and *S. pennelli* cross F2 population [60]. However, less overlap was observed when comparing our data with the germination QTLs from an *S. esculentum* and *S. pimpinellifolium* (accession LA722) back cross (BC) 1 population [64]. This demonstrates the influence of different parental allelic variation on QTL detection, and the benefit of using multiple parents for QTL mapping [63].

QTLs related to germination from seeds developed in different maternal environments have been identified in a lettuce RIL population [65]. QTLs’ underlying maternal environmental effects on dormancy and germination have also been studied in Arabidopsis [66]. A study on a combination of near-isogenic lines (NIL) and mutant Arabidopsis lines showed that the ABA-related genes DOG1 and cyp707a1 mediate the influence of various developmental conditions on dormancy and germination [32]. In addition, the expression of FLOWERING LOCUS T in silique tissue was found to convey the effect of maternal growth temperatures on seed germination [34].

Possible mechanisms of the trans-generation “stress imprint” include the accumulation of resistance-conferring proteins, the induction of stress response transcription factors and epigenetic modifications [67, 68]. These environmentally induced factors are preserved in the dry seed and influence germination. Similarly, during seed maturation and desiccation changes in metabolic gene transcripts, proteins and metabolites that are preserved in dry seeds are functionally involved in modulating germination [9, 69–72]. The high number of metabolite-germination correlations found suggests that primary metabolites could play a role in mediating the effect of maternal growth conditions on seed germination.

### Metabolic response to salinity

We observed little alteration in seed metabolites in response to stress; only 1% of the RMC comparisons between SDS and SDF showed statistical significance. This is in contrast to previous studies on the effect of salinity on tomato pericarp metabolites [73, 74] and of temperature and nitrogen on Arabidopsis seeds [75]. Nevertheless, it has been shown that different parts of the plant, even different parts of the fruit, can have different metabolic responses to salt stress [73]. Supported by the higher broad-sense heritability found in seed metabolic traits, as opposed to fruit metabolic traits [25], these results further indicate the highly preserved traits amid environmental perturbations in seeds compared to fruit. Of the RMC comparisons that did change in response to the maternal environment, some were found in M82 seeds and a number were found in ILs seeds. In M82, all metabolites displaying increased RMC in SDS, compared to SDF, clustered to ms1 (such as methionine, GABA, cysteine, asparagine and ferulate). The accumulation of ms1 metabolites in SDS was found also in the few ILs that, like M82, had reduced germination in SDS, whereas metabolites with a decreased abundance in response to salinity in these ILs were clustered to ms2. ILs with improved germination in SDS had the opposite pattern, an increase in metabolites from ms2 and a decrease in ms1. Taken together, these results demonstrate that changes in the metabolite sets are accompanied by differences in germination and validate our hypothesis of a metabolic imprint in seeds conferring effects on germination.

### Metabolic relations to germination

The most remarkable finding from the data analysis was that the metabolic network could be divided into two opposite sets, ms1 and ms2, which show complete correspondence to the germination measures. All metabolites with positive correlations to germination were in ms2, while all metabolites with negative ties were in ms1. The division of metabolites into the two balanced sets, based on positive correlations to metabolites within the same set, was conserved throughout the four examined networks (salinity conditions and seasons). The unequivocal relation between ms1 and ms2 and germination in the additional analysis methods emphasizes its biological significance.

Correlations do not necessarily imply a cause and effect relationship, but it may be suggested that the levels and proportions of some metabolites in the seed affect processes required for germination. Allen et al. [76] suggested that metabolites may play a regulatory role over gene expression, resulting in correlations between metabolites and between metabolites and gene expression. Alternately, metabolite accumulation or depletion can be an outcome/byproduct of processes the seed underwent, which also affects germination ability; for example, it may represent a defined metabolic status of different levels of after-ripening or dormancy. Nevertheless, we will attempt to discuss the possible link between some specific metabolites and germination.

Amino acids of the central network module were negatively correlated to germination. Free amino acids are known to act as substrates for energy production in early germination stages [10, 13, 77–79], hence the results were not expected. They also provide nitrogen resources and amino acids readily available for protein synthesis, which is required for germination [2, 80]. Nevertheless other studies that harnessed the balance of given amino acids, specifically in the seeds, showed that accumulation of metabolites such as methionine, lysine and GABA can lead to decreased germination [13, 81, 82]. External applications of several amino acids also significantly reduce germination of lettuce and broomrape, and have been suggested as a means of biological control [83, 84]. A possible explanation for the phenomenon is that higher levels of specific amino acids could deplete the level of other metabolites important for the advancement of germination. For example, in KD mutants where lysine catabolism is blocked by siRNA, the mutants display lysine accumulation and deficient germination [13], in accordance with the negative correlation between lysine and germination found here. Lowered levels of TCA cycle metabolites in the KD mutant suggest that lysine accumulation interferes with germinating seeds’ energy production via the TCA cycle. Alternatively, a regulatory link between metabolites and gene expression might also be hypothesized. For example, in the seed-specific gad1 Arabidopsis transgenic lines that accumulate GABA, the major regulatory gene of germination, DELAY OF GERMINATION1 (DOG1), was among the few genes significantly induced [82]. Recent studies have found DOG family genes to be involved in the germination and dormancy response of seeds to maternal environment [32].

Methionine was one of the metabolites most negatively and strongly correlated to germination in our study. It has been found to strongly inhibit germination in lettuce [83]. In some studies, high methionine levels in seeds were found to be a limiting factor for the synthesis of sulfur-rich proteins [81], inhibiting germination. Only a few successful attempts at increasing methionine content in storage protein led to normal germination [85] or mild reduction [86]. In Arabidopsis, de novo methionine biosynthesis is required for germination [87]. When the methionine biosynthesis pathway was blocked, germination was greatly reduced and seedlings did not reach establishment. A possible mechanism for this negative correlation is the inhibitory effect that S-adenosylmethionine (AdoMet), a product of methionine catabolism, has on cystathionine γ-synthase, an enzyme in the early stages of its biosynthesis pathway [88]. Furthermore, AdoMet induces precursor allocation to other pathways [2], thereby perhaps reducing the production of essential metabolites, such as methylation components and ethylene that are products of the methionine pathway. While the relation between the alteration in metabolite level and the induction of regulatory genes needs to be elucidated, these studies demonstrate the inhibitory effect of unbalanced amino acid metabolism in seeds on germination.

Notably, the N-containing metabolites dopamine and urea were, in ms2, positively correlated to germination. In plants, dopamine is part of catecholamine biosynthesis from tyrosine (plantCyc: [41]). It has been found in low amounts in tomato and in other plant species [89, 90]. Catecholamines and dopamine increase stress tolerance, which can be attributed to dopamine’s high antioxidant potency [90, 91]. In addition, it was found to improve rice germination under salinity [92]. Indeed, in this study, dopamine was 3.9 times higher in well-germinating seeds than in non-germinating seeds. That said, the mechanisms underlying a dopamine-positive association with germination remains unclear. Urea is a product of amino acid catabolism, and the effect it has on germination has not been directly studied to our knowledge.

Accumulated amino acids could also reflect the degradation of proteins during development or post-harvest, leading to defective germination. Controlled deterioration and post-harvest stress studies have shown an increase in protein degradation and increased levels of amino acids and their leakage accompanied by the loss of germination vigor [93–95]. Nevertheless protein degradation would be accompanied by a general increase in free amino acids, not in specific amino acids.

Simple sugars, although expected to support germination as energy sources, were also negatively correlated to germination. Carbohydrate degradation, accompanied by the accumulation of simple sugars, has been linked to poor germination influenced by stress and seed aging [96, 97]. Glucose utilization has been suggested as a biomarker for seed vigor, as its levels were found to be sensitive and to precede any visible growth change in response to seed aging [98]. Glucose was one of the metabolites with the highest contribution to the separation of non-germinating seeds and germinating seeds in PCA, and all simple sugar levels were significantly higher in non-germinating seeds than in all other germination levels. In line with our results, glucose has been shown to delay germination [99], suppress starch degradation in lupine [100] and rice [99, 101] and starch mobilization [11], as well as induce ABA production in germinating seeds [11, 101]. The latter likely accounts for the observed inhibition of seed germination [12]. Interestingly, in mutant Arabidopsis seeds unable to remove ABA inhibition during imbibition, ABA signaling was overruled by exogenous sucrose [102], whose endogenous levels in the present work were found to have a positive correlation to germination.

Galactinol levels were significantly higher in germinating seeds and contributed to their separation from non-germinating seeds in PC2. Galactinol is related to the raffinose family of oligosaccharides and a precursor in raffinose biosynthesis from galactose (KEGG). It has been shown to increase in Arabidopsis seeds developed under high light intensity, and was correlated to seed longevity [75]. Transcripts of Galactinol synthase accumulate prior to desiccation in tomato and maize seeds [103, 104]. Raffinose and similar compounds have been found to contribute to seeds as energy sources for early germination [10]. Thus, it is likely that seeds with higher galactinol levels could provide the germination process with immediate, easily mobilized energy. In addition, as a product of galactose, galactinol could accumulate due to the enhanced cell wall degradation of which galactose is a byproduct. The metabolite with the strongest positive association to germination in this study was threonate. Threonate was more than four-fold higher in well-germinating plots than in non-germinating plots. A product of ascorbate, threonate also made a high contribution to the variance in PC2 and had a strong positive correlation to germination percent. To the best of our knowledge, no previous study has addressed the effect of threonate on seeds and germination, although it has been found in seeds [105, 106]. Together with threonate, malate also displayed a positive correlation to germination. A possible way this metabolite can enhance germination is by “Perl’s pathway”, first suggested by Perl [107]. In this pathway, malate and aspartate, both positively related to germination, are utilized for ATP production in the early stages of germination [10, 108].

### Outstanding ILs

Several ILs stand out with a high number of significant differences in various traits.

IL1-4-18 displayed many QTLs of FC in RMC in response to salinity. It also had a QTL for improved germination rate (Additional File 2) and higher seed weight in SDS. The corresponding genomic segment was identified as a QTL affecting tolerance to germination under salinity [60].

IL2-1-1 displayed many metabolic differences compared to M82 in both SDS (Additional file 2) and SDF, accompanied by several FC-QTLs. IL2-1, whose introgression segment completely contains that of IL2-1-1, also showed numerous s-QTLs and f-QTL with overlap in specific metabolite QTLs. It had an improved germination percent in SDS vs. SDF and a higher percent than M82 in both conditions. Under both conditions, metabolites of ms2 were higher in these ILs than in M82, and metabolites from ms1 were lower. Also, in SDS vs. SDF, metabolites of ms2 and ms1 displayed an increase and a decrease, respectively, further validating how the two groups relate to germination in specific ILs. The corresponding region to IL2-1 and part of IL2-1-1 have previously been identified to have a germination QTL [60].

IL3-2 and IL3-4 share many characteristics, as well as morphological and physiological traits. They also shared several QTLs of leaf antioxidant content in response to salinity [74], as well as similar metabolite differences, compared to M82, in dry seeds [25]. Eshed and Zamir [18], in the earliest report of the IL population, also demonstrated similarities in IL3-2 and IL3-4 in their reproductive traits, fruit mass, Brix°, yield and Brix-yield; however, they differ in vegetative weight, according to both Eshed and Zamir and the data collected for this report. Foolad [109] found salinity tolerance during germination QTLs using RFLP markers corresponding with the genomic span of IL3-2 and IL3-4. Both ILs had orange fruit, with color further reduced by salinity, and a lower number of fruit than M82 in both treatment regimes, as well as some reduction in fruit weight. Although IL3-2 had higher fruit Brix° than M82, both ILs had considerably lower yield measures in all conditions (Perelman, unpublished observations). The number of mature seeds per fruit was reduced by about half in these ILs compared to M82, but with no significant difference in seed weight between lines or growth conditions. In contrast to M82, IL3-4 maintained and even improved its seed germination percent in SDS (IL3-2 had improved germination, but did not meet the significance threshold). Many s-QTLs and FC-QTLs in IL3-2 and IL3-4 stand out in opposition to the few QTLs in SDF. Metabolites that increased by salinity in these ILs belonged to ms2, and decreased metabolites belonged to ms1, supporting a relation between seed metabolic balance in response to salinity and germination. The consistent similarity and proximal genomic location may suggest a duplication of a chromosomal segment, which evolved paralogous genes.

## Conclusions

In tomato, seed weight is genetically governed and maintained despite moderate stress conditions at the expense of seed number. The hypothesis that seed weight is conserved to maintain seed quality is supported by the overall weak effect of the environment on seed metabolism and germination observed. Metabolite profiles were found to have an unequivocal relation to germination. We demonstrate that it is not the accumulation of one metabolite but a metabolic balance between groups of metabolites that mediates germination. Irrespective of the origin or effect of primary metabolites on germination, the multiple analyses demonstrate that there is a relationship between the dry seed metabolic profile and the germination ability of the seeds. This suggests a potential for determining a metabolic signature that can be used for assessing and possibly predicting the germination vigor of seed batches.

## Methods

### Plant material and growth conditions

A population of 72 introgression lines (ILs), each containing a well-defined chromosome segment of a wild tomato species, *S. pennellii*, in the background of domesticated tomato *L. esculentum* cultivar M82 [18], was used. The ILs and their control line M82 were grown in a field experiment under fresh water (EC = 1.5 dS/m) and saline water (EC = 6 dS/m) irrigation regimes, during two consecutive seasons. Each line had five replicate plots under each treatment, organized in a split plot (salinity in main plots, genotypes in sub-plots) random block design. The experiment was conducted in the Ramat-Negev R & D Facility in the summers of 2010 (season I) and 2011 (season II). For each field plot, seeds from four plants were extracted, pooled, dried and stored at room temperature. Seed samples were used for seed morphology examination, germination tests and metabolite profiling by GC-MS.

### Seed weight and count

Average seed weight was calculated based on seed count and the exact seed weight used for metabolite extraction. In season I, seeds from two ripe tomato fruits from each plot were sorted according to maturity and counted. Mature seeds were defined as seeds with a hard and slightly hairy seed coat, with typical seed size, thickness and color. Other seeds, such as greatly deformed or aborted seeds, were counted separately. Aborted seeds were identified by the lack of a hard seed coat and very thin and light-colored seeds. Dissection of seeds of this description under a binocular microscope showed they contained no embryo. The aborted seeds constituted the great majority of non-mature seeds.

### Germination tests

Mature seeds from the season I field experiment, from both growth conditions, were examined for germination vigor. The seeds were surface-sterilized in 2% bleach for 10 min and rinsed three times in autoclaved tap water. Seeds were germinated on three layers of filter paper saturated with water, and the excess water was drained. The experiment was conducted in a growth room with 16 h of light, 8 h of dark, and a temperature range of 20–25 °C. Each day, the number of seeds that had germinated was recorded. We then calculated the percentage of seeds that germinated and the number of days until 50% (T50) of those seeds germinated, and we described the variation in the number of days to germination within plates using the standard deviation of the average days to germination within each plate. There were at least three replicate plates for each line and condition, with 50 seeds per plate.

### Metabolite quantification: sample extraction and derivatization

Mature dry seeds from each four plant plot in the field experiments were pooled and extracted using a standard lab protocol. Approximately 50 mg of seeds were ground using a pre-cooled tissue lyzer (400MM, Retsch, Germany). In season I, metabolites were extracted from each experimental plot separately, and in season II, seeds of the five replicate plots were pooled for extraction and analysis. Seed powder was kept in liquid nitrogen from grinding until complete immersion in 1 ml of pre-cooled extraction mix. The mix contained a ratio of 2.5:1:1 (v/v) of methanol, DDW and chloroform, respectively. A ribitol (Fluka Analytical) internal standard was dissolved in DDW to a final concentration of 3.8 mg/ml prior to mixing with chloroform and methanol. In addition to the mix, 100 μl of pre-cooled methanol was added to the sample. The sample was homogenized by vortex and then put in a shaker (1000 rpm) at room temperature for 10 min, followed by 10 min in a sonication bath. Next, samples were centrifuged for 4 min at 14,000 rpm in an Eppendorf 5417 R centrifuge. The supernatant was transferred to a new test tube and an additional 300 μl of chloroform and 300 μl of water were added. Samples were vigorously vortexed and then centrifuged for 2 min at 14,000 rpm. The top phase was separated and 100 μl was dried in a vacuum using an Eppendorf concentrator plus for GC-MS analysis. The dried samples were derivatized prior to GC-MS analysis. A volume of 40 μl of 20 mg/ml metoxyaminehydrochloride (Sigma-Aldrich®) in pyridine was added to the dried pellet and incubated for 2 h in an orbital shaker at 1000 rpm at 37 °C. An N-methyl-N-(trimethylsilyl) trifluoroacetamide (MSTFA) mix containing time standards (Alkanes for Thermo GC-MS or fatty acid methyl-esters (FAMEs) for Agilent GC-MS, 1.2 μg of each FAME) in a volume of 77 μl was added to each sample and then incubated for an additional 30 min in an orbital shaker at 37 °C. Finally, 1 μl of the derivatized sample was injected into the GC-MS machine.

### Metabolite profiling: CG-MS-qTof analysis

All sample analyses and metabolite quantification were achieved by using a GC-MS system from Waters Ltd, which consisted of an Agilent 7683B series autosampler and injector (Agilent Technologies), an Agilent 7890A gas chromatograph, and a Xevo™ QTof mass spectrometer. Sample volumes of 1 μL were injected onto the GC column with a splitless and split mode ratio of 10:1. GC was performed on a 30-m VF-5 ms column with 0.25-mm i.d. and 0.25-μm film thickness +10 m EZ-Guard (Agilent). Inlet injection temperature was set at 280 °C. The carrier gas used was helium set at a constant flow rate of 1 ml/min. The temperature program was 1 min isothermal heating at 70 °C, followed by a 10 °C/min oven temperature ramp to 350 °C, followed by a final 5 min of heating at 350 °C. The transfer line temperature was set to 310 °C. The makeup gas for APGC was nitrogen from tanks at 110 psi. The MS system used was Xevo™ QTof (Waters MS Technologies, Manchester, UK) operating in a positive ion mode. The MS source used was API. Analysis calibration was obtained using a siloxane mass of 281.05 Da m/z for lock mass calibration to ensure accuracy and reproducibility, with a scan time of 0.5 s, a cone voltage of 35 V and a capillarity of 2 kV. For the samples, the following parameters were used: capillary voltage: +3.1 kV; sampling cone voltage: 14 V (season II 35 V); extraction cone voltage 4 V; source temperature: 150 °C; desolvation temperature: 45 °C; cone gas flow: 11 L/h; desolvation gas flow: 400 L/h; and collision energy: 6 V. For the MS/MS spectra, collision energies were set from 10 to 30 V. Detection modes for MS and MS/MS were: scan ranges from 50–700 m/z. Scan time was 0.08 s.

Initial peak identification was performed using a Thermo GC-MS, since structural libraries for analyses of the Waters machine output are limited. The GC-MS system consisted of an AS 3000 autosampler, a TRACE GC ULTRA gas chromatograph, and a DSQII quadrupole mass spectrometer (Thermo-Fisher Scientific Ltd). GC was performed on a 30-m VF-5 ms column with 0.25-mm i.d. and 0.25-μm film thickness +10 m EZ-Guard (Agilent). The gradient of injection temperature (PTV) was from 60 °C to 300 °C at 14.5 °C/s. The transfer line was set to 300 °C, and the ion source adjusted to 250 °C. The carrier gas used was helium set at a constant flow rate of 1 ml/min. The temperature program was 1 min isothermal heating at 70 °C, followed by a 1 °C/min oven temperature ramp to 76 °C, followed by a 6 °C/min oven temperature ramp to 350 °C, and a final 5 min of heating at 350 °C. The mass spectrometer was tuned according to the manufacturer’s recommendations using tris-(perfluorobutyl)-amine (CF43). Mass spectra were recorded at 8 scans per second with a mass-to-charge ratio of 70 to 700 scanning range, with electron energy of 70 eV. Spectral searching and peak identification utilized the National Institute of Standards and Technology (NIST, Gaithersburg, USA) algorithm incorporated in the Xcalibur® data system (version 2.0.7) against RI libraries downloadable from the Max Planck Institute for Plant Physiology in Golm (http://gmd.mpimp-golm.mpg.de/).

### Metabolite annotation

Initial metabolite annotation was carried out with samples run in the Thermo GC-MS using Xcalibur® software and the “var” and “mainlib” libararies in the NIST software. The initial annotation list was compared to samples run in the Waters GC-MS-Tof using MassLynxTM software and verified using ChemSpider structural elucidation software online ([110]; http://www.chemspider.com). For some metabolites, chemical standards were examined for verification.

### Data treatment (normalization) and statistical analysis

Metabolite content quantification was achieved using MarkerLynx (Waters Ltd.) software. The height of selected mass peaks was extracted from the program output by a specifically designed R script. Peak height was normalized according to seed weight before extraction, the sum of peaks in the sample, and for each metabolite, to the median of value of the specific metabolite across samples of every run (block) separately. The complete normalized dataset is available in Additional File 1: Table S1. In order to improve normality, the data was log transformed prior to statistical tests. Calculation of descriptive statistics, t-tests and correlation analysis was performed in R statistical and graphics software v2.14.0 (R core team).

In an attempt to maximize the discovery power of the statistical test, but avoid false discovery, a permissive Bonferroni correction threshold was used by applying the Bonferroni correction for the square-root of the number of comparisons (√n) on *α* = 0.05 (*p* < 0.05, Bcp).

Prior to correlation analysis, missing values were completed with the “completeObs” function of the R PCA package [111, 112]. For correlations, the FDR equivalent of *p* = 0.05 was used as a threshold. Metabolite and germination correlation were calculated using Spearman’s rank correlation [40]. Node communities were calculated using the Walktrap algorithm in the R “igraph” package [113]. Significant correlations were transformed into a graphic network using Cytoscape v3.1.1.

Principal component analysis (PCA) and K-means clustering (KMC) were obtained using tMeV software v4.8 ([114]; http://www.tm4.org).

## Additional files

Additional File 1: Table S1. Normalized full metabolic dataset. **Table S2.** Seed weight in response to salinity for both seasons. **Table S3.** Putative QTLs for maturation percent. **Table S4.** Putative QTLs for RMC in SDF. **Table S5.** Putative QTLs for RMC in SDS. (XLSX 882 kb)
Additional File 2: QTL Map of co-localized metabolite and germination QTLs in SDS. (PDF 230 kb)
Additional File 3: Correlation networks of 2010 for SDF. (PDF 545 kb)
Additional File 4: Correlation networks of 2010 for SDS. (PDF 786 kb)

## Abbreviations

ABA: Abscisic acid
AdoMet: S-adenosylmethionine
Bc: Bonferroni correction
BC: Back cross
DOG1: DELAY OF GERMINATION1
EC: Electrical conductivity
FC: Fold-change
FDR: False discovery rate
GABA: γ-amino butyric acid
GC-MS: Gas chromatograph-mass spectrometry
IL: Introgression lines
KMC: K-means clustering
MMP: Monomethylphosphate
ms1: Metabolite set 1
ms2: Metabolite set 2
NIL: Near-isogenic lines
PCA: Principal component analysis
QTLs: Quantitative trait loci
RIL: Recombinant inbreed line
RMC: Relative metabolite content
SDF: Seeds Developed on plants grown in Fresh water
SD-plate: Standard deviation of germination day within an experimental plate
SDS: Seeds Developed on plants grown in Saline irrigation
TCA: Tricarboxylic acid
