# Supplementary material for: Environmental and genetic effects on tomato seed metabolic balance and its association with germination vigor
Source: BMC Genomics. 2016 Dec 19;17:1047. doi: 10.1186/s12864-016-3376-9 (PMC5168813; doi:10.1186/s12864-016-3376-9)
Supplement: Additional File 2: — QTL Map of co-localized metabolite and germination QTLs in SDS. (PDF 230 kb) [file 12864_2016_3376_MOESM2_ESM.pdf]

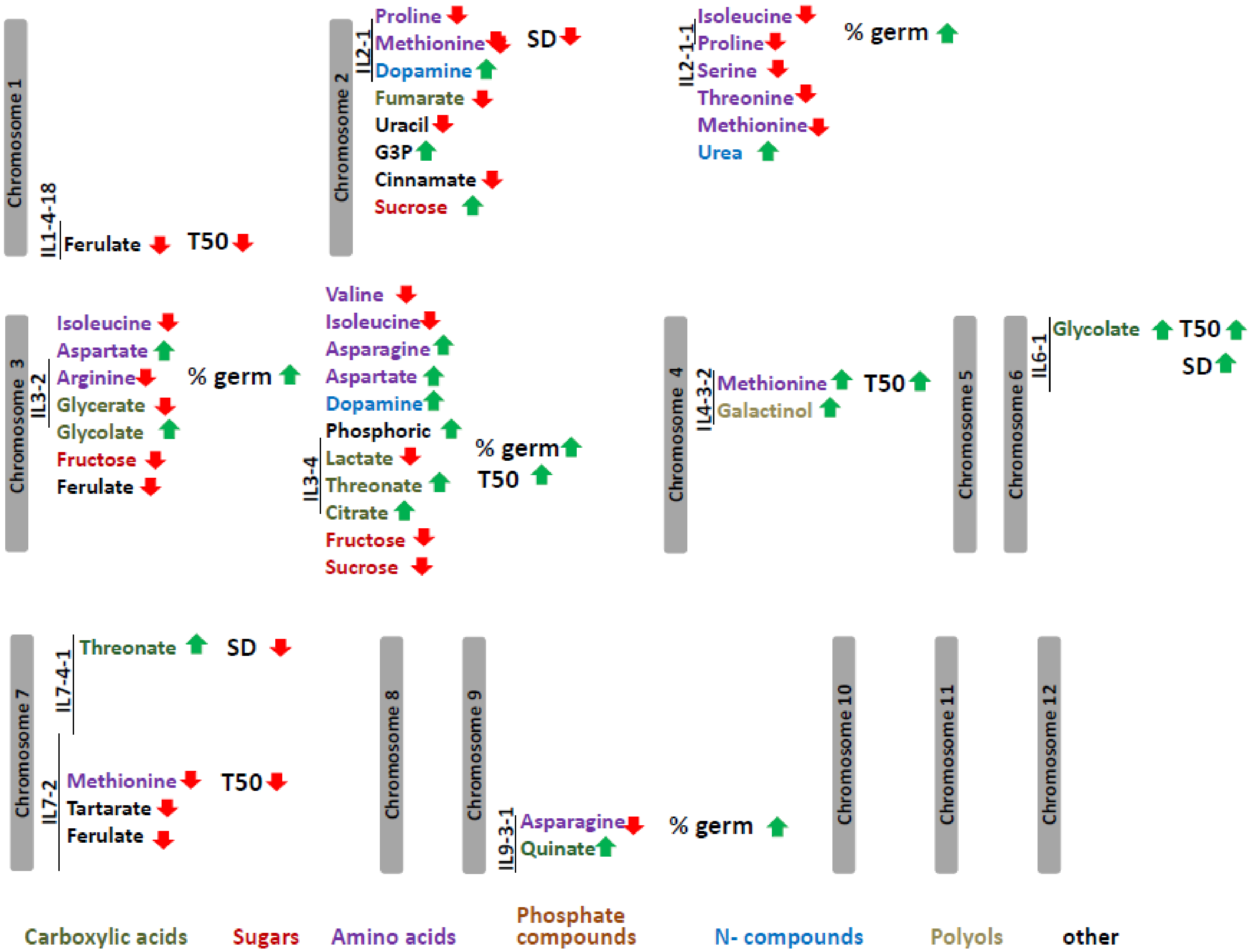

Figure S2- QTL map of metabolic and germination co-localized QTLs in SDS

Metabolites which had significant ($p<0.05$, Bc) differences in SDS as well as significat changes in germination in an IL compared to M82 are noted in parallel to the genomic location of the introgression segment. Colors represent metabolite class, as indicated.
