# Supplementary material for: Environmental and genetic effects on tomato seed metabolic balance and its association with germination vigor
Source: BMC Genomics. 2016 Dec 19;17:1047. doi: 10.1186/s12864-016-3376-9 (PMC5168813; doi:10.1186/s12864-016-3376-9)
Supplement: Additional File 3: — Correlation networks of 2010 for SDF. (PDF 545 kb) [file 12864_2016_3376_MOESM3_ESM.pdf]

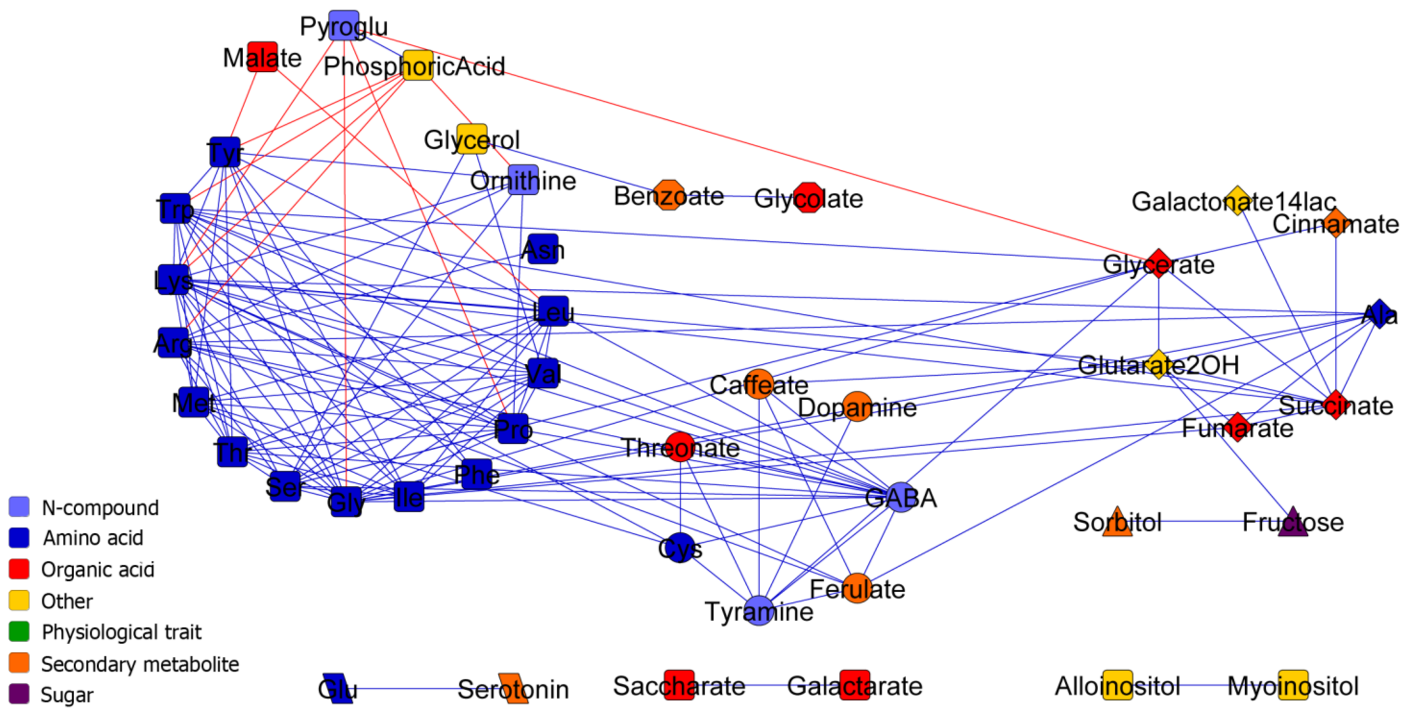


**Figure S1A- Correlation network of metabolites of SDF in season II**

Nodes depict metabolites and germination measures. Metabolite nodes were arranged according to Walktrap communities (also indicated by node shape), and colored by chemical class, as indicated. Edges represent significant (p<0.05, FDR; |r|>0.4) correlations. Each community was separated according to positive (blue) and negative (red) correlations.
