# Supplementary material for: Environmental and genetic effects on tomato seed metabolic balance and its association with germination vigor
Source: BMC Genomics. 2016 Dec 19;17:1047. doi: 10.1186/s12864-016-3376-9 (PMC5168813; doi:10.1186/s12864-016-3376-9)
Supplement: Additional File 4: — Correlation networks of 2010 for SDS. (PDF 786 kb) [file 12864_2016_3376_MOESM4_ESM.pdf]

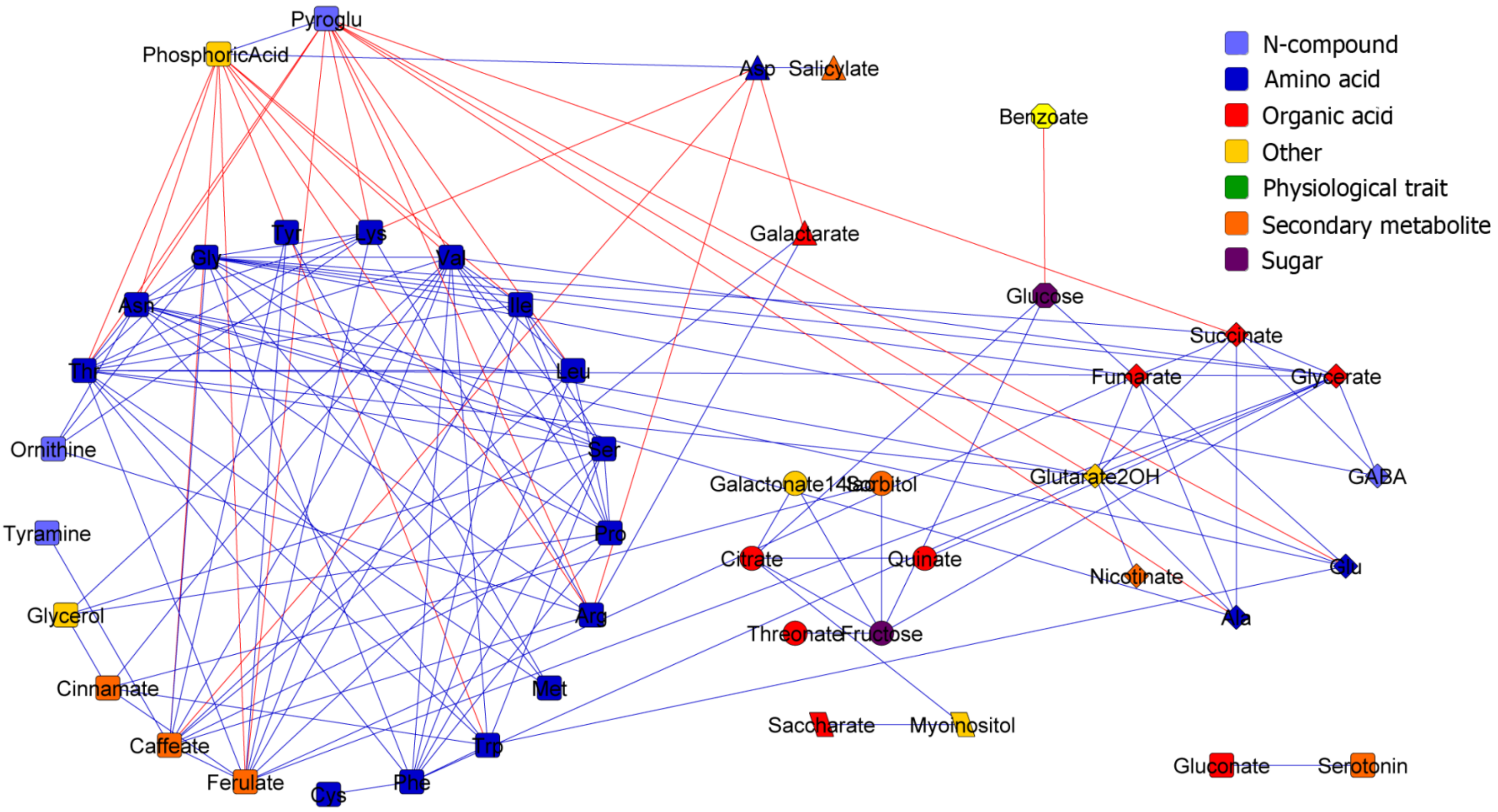

Figure S1B- Correlation network of metabolites of SDS in season II
Nodes depict metabolites and germination measures. Metabolite nodes were arranged according to Walktrap communities (also indicated by node shape), and colored by chemical class, as indicated. Edges represent significant ($p<0.05$, FDR; $|r|>0.4$) correlations. Each community was separated according to positive (blue) and negative (red) correlations.
